# Omentin-1 inhibits the development of benign prostatic hyperplasia by attenuating local inflammation

**DOI:** 10.1186/s10020-024-00805-y

**Published:** 2024-03-22

**Authors:** Yi-Yi Wang, Guo-Qiang Zhu, Kun Xia, Hong-Bo Zeng, Yun-Hui He, Hui Xie, Zhen-Xing Wang, Ran Xu

**Affiliations:** 1 Department of Orthopedics, Movement System Injury and Repair Research Center, Xiangya Hospital, Central South University, Changsha, 410008 Hunan China; 2 Department of Urology, The Second Xiangya Hospital, Central South University, Changsha, 410011 Hunan China; 3 Hunan Key Laboratory of Angmedicine, Changsha, 410008 Hunan China; 4 National Clinical Research Center for Geriatric Disorders (Xiangya Hospital), Changsha, 410008 Hunan China; 5 Department of Orthopedics, People’s Hospital of Ningxia Hui Autonomous Region, Yinchuan, 750000 Ningxia China

**Keywords:** Benign prostatic hyperplasia, Omentin-1, Intelectin-1, Inflammation, Prostate epithelial cells, Macrophages

## Abstract

**Background:**

Benign prostatic hyperplasia (BPH) is a prevalent disease affecting elderly men, with chronic inflammation being a critical factor in its development. Omentin-1, also known as intelectin-1 (ITLN-1), is an anti-inflammatory protein primarily found in the epithelial cells of the small intestine. This study aimed to investigate the potential of ITLN-1 in mitigating BPH by modulating local inflammation in the prostate gland.

**Methods:**

Our investigation involved two in vivo experimental models. Firstly, ITLN-1 knockout mice (*Itln-1*^*−/−*^) were used to study the absence of ITLN-1 in BPH development. Secondly, a testosterone propionate (TP)-induced BPH mouse model was treated with an ITLN-1 overexpressing adenovirus. We assessed BPH severity using prostate weight index and histological analysis, including H&E staining, immunohistochemistry, and enzyme-linked immunosorbent assay. In vitro, the impact of ITLN-1 on BPH-1 cell proliferation and inflammatory response was evaluated using cell proliferation assays and enzyme-linked immunosorbent assay.

**Results:**

In vivo, *Itln-1*^*−/−*^ mice exhibited elevated prostate weight index, enlarged lumen area, and higher TNF-α levels compared to wild-type littermates. In contrast, ITLN-1 overexpression in TP-induced BPH mice resulted in reduced prostate weight index, lumen area, and TNF-α levels. In vitro studies indicated that ITLN-1 suppressed the proliferation of prostate epithelial cells and reduced TNF-α production in macrophages, suggesting a mechanism involving the inhibition of macrophage-mediated inflammation.

**Conclusion:**

The study demonstrates that ITLN-1 plays a significant role in inhibiting the development of BPH by reducing local inflammation in the prostate gland. These findings highlight the potential of ITLN-1 as a therapeutic target in the management of BPH.

**Supplementary Information:**

The online version contains supplementary material available at 10.1186/s10020-024-00805-y.

## Highlights

1. ITLN-1 knockout mice presented heavier prostate weights and higher inflammation levels in the prostate than their WT littermates.
2. In a testosterone propionate-induced BPH mouse model, adenovirus-mediated ITLN-1 overexpression led to a reduction in prostate weight and local inflammation.
3. ITLN-1 can inhibit prostate epithelial cell proliferation by suppressing macrophage-mediated inflammatory responses.
4. Targeting ITLN-1 could be an effective therapeutic approach for preventing or treating BPH in elderly men.

**Supplementary Information:**

The online version contains supplementary material available at 10.1186/s10020-024-00805-y.

## Introduction

Benign prostatic hyperplasia (BPH) is a prevalent age-related condition among elderly men, characterized by prostate enlargement, leading to urinary complications and a reduced quality of life (Zeng et al. 2022). BPH affects approximately 25% of men aged 50 to 59 years, and 50% of men aged over 60 years, with the prevalence increasing with age (Wei et al. 2005). Currently, the management of BPH often involves pharmacological interventions and, in severe cases, surgical procedures (Miernik and Gratzke 2020; Zitoun et al. 2020). However, these treatments can be costly and may have undesirable side effects, including sexual dysfunction and decreased libido (Giuliano et al. 2013; Srinivasan and Wang 2020). Consequently, there exists a pressing need for novel therapeutic strategies to impede or decelerate the progression of BPH.

Recent research has highlighted the significant roles of metabolic syndrome and chronic inflammation in the development and progression of BPH (Chang et al. 2019; Park et al. 2022; Passos et al. 2021). Chronic prostatic inflammation, in particular, is a key factor in the pathogenesis of BPH (Jin et al. 2023; Lin et al. 2022). It is associated with elevated prostate symptom scores and increased prostate volume. Chronic inflammation in the prostate can lead to an overproduction of growth factors, such as tumor necrosis factor-alpha (TNF-α) and interleukin-6 (IL-6), causing excessive prostate cell proliferation and remodeling (Vignozzi et al. 2013). Furthermore, emerging evidence highlights the significance of metabolic syndrome in the progression of BPH. A prospective study involving 78 BPH patients, which found that those with metabolic syndrome had significantly higher median annual growth rates in both total prostate volume and transitional zone volume compared to patients without metabolic syndrome (Ozden et al. 2007). This association highlights the intricate link between metabolic health and prostatic diseases, further emphasizing the importance of considering metabolic factors in BPH pathogenesis and management. It also underscores the need for developing new treatment strategies that target these underlying risk factors.

Omentin-1 (Intelectin-1, ITLN-1), an adipokine with anti-inflammatory properties (Rao et al. 2018; Zabetian-Targhi et al. 2016; Zhao et al. 2022), emerges as a potential therapeutic agent. ITLN-1 secreted by human visceral fat and the small intestine, and solely by the small intestine in mice (Nonnecke et al. 2022; Watanabe et al. 2017). ITLN-1 is inversely associated with metabolic syndrome, a known risk factor for BPH (Watanabe et al. 2017; Zabetian-Targhi et al. 2016). Its role in reducing pro-inflammatory cytokines and increasing anti-inflammatory suggests its potential in regulating prostate inflammation. Our previous clinical serologic study has indicated a possible association between decreased levels of omentin-1 and BPH in patients (He et al. 2020), implying its involvement in prostate cell growth regulation.

Despite documented anti-inflammatory, anti-obesity, and anti-diabetic properties observed in various populations (Tan et al. 2010; Watanabe et al. 2017), the precise role of ITLN-1 in the pathogenesis of BPH remains unclear. This study aimed to clarify the role of ITLN-1 in BPH pathogenesis and its therapeutic implications. Utilizing ITLN-1 knockout mice (*Itln-1*^*−/−*^) and adenovirus-mediated ITLN-1 overexpression models, we explored the effects of ITLN-1 on prostate inflammation and cell proliferation. In vitro and in vivo studies demonstrated that ITLN-1 was able to suppress macrophage-mediated inflammatory responses. Our findings suggest that omentin-1 may represent a promising therapeutic target for the treatment of BPH.

## Results

### Elder Itln-1 knockout mice exhibit spontaneous BPH

**Fig. 1:**
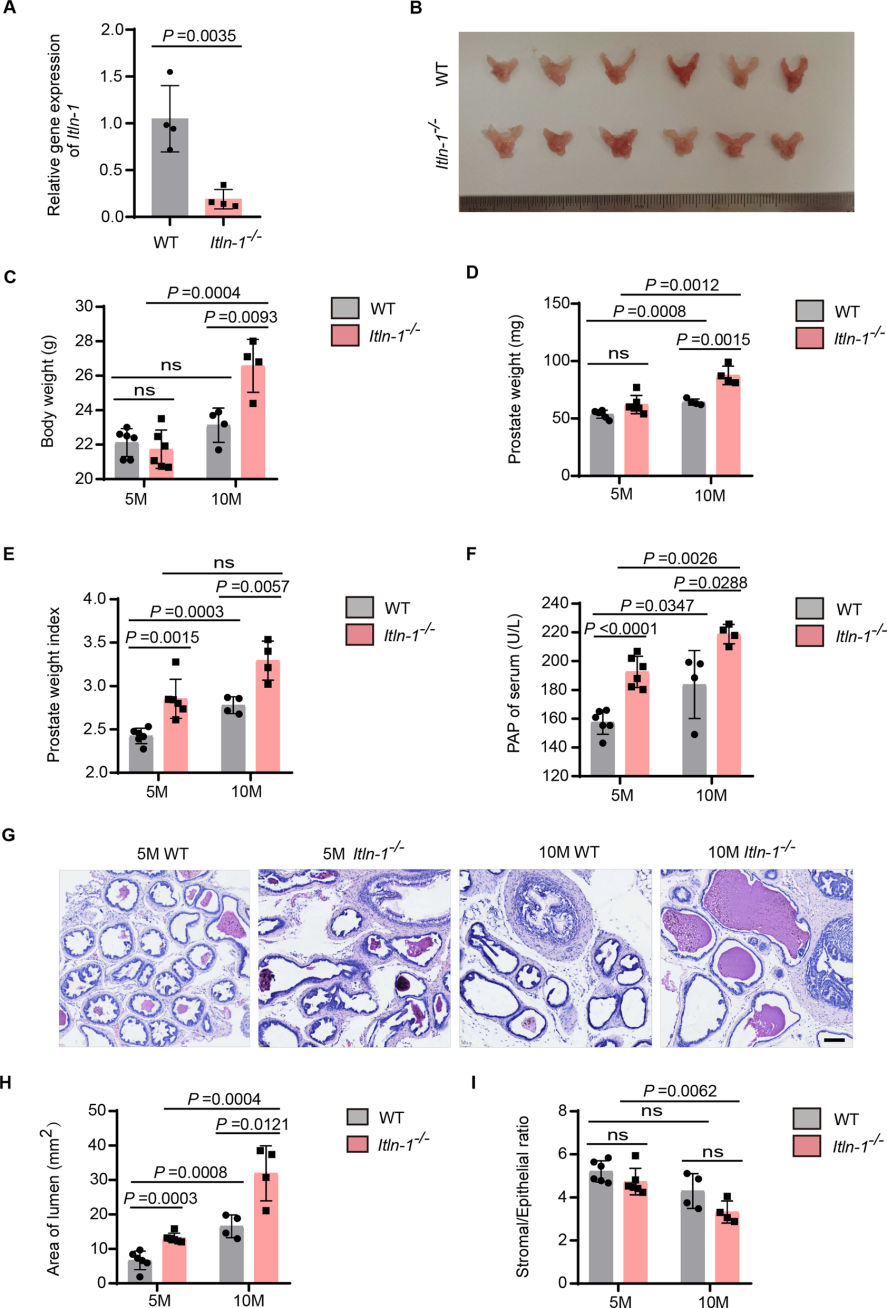
Elder *Itln-1* knockout mice exhibit spontaneous BPH. **A** Relative gene expression levels of *Itln-1* in the small intestine of 5-month-old wild type (WT) mice and *Itln-1*^*−/−*^ mice. n = 4, t-test*.* **B** Gross anatomy of prostate tissues from 5-month-old WT and *Itln-1*^*−/−*^ mice. **C**–**F** The body weight (**C**), prostate weight (**D**), prostate index (**E**), and serum levels of PAP (**F**) in 5-month-old (5 M) or 10-month-old (10 M) WT and *Itln-1*^*−/−*^ mice, respectively. n = 6 for 5-month-old mice, n = 4 for 10-month-old mice, two-way ANOVA. The prostate index was calculated as the ratio of prostate wet weight (mg) to the body weight (g) of the mice. **G** Representative images of H&E staining of prostate tissues from WT and *Itln-1*^*−/−*^ mice, observed under 200-fold magnification. Scale bar: 50 μm. **H**, **I** Area of lumens (**H**) and stromal/epithelial ratio (**I**) of WT and *Itln-1*^*−/−*^ mice, respectively. n = 6 for 5-month-old mice, n = 4 for 10-month-old mice, two-way ANOVA. The values of the individual statistical significances and statistical comparisons were indicated in the figure

To investigate the potential role of omentin-1 in BPH development, we successfully generated the *Itln-1* knockout (*Itln-1*^*−/−*^) mice as our previously reported (Rao et al. 2018). Due to the *Itln-1* was specifically expressed in the small intestines of mice, the depletion of *Itln-1* in their small intestines of *Itln-1*^*−/−*^ mice was confirmed by reverse transcription quantitative PCR (RT-qPCR). The results showed an approximately 82% decrease of *Itln-1* in *Itln-1*^*−/−*^ mice compared to wild type (WT) mice (*P* = 0.0035; Fig. 1A). We then evaluated whether omentin-1 deficiency resulted in prostate hyperplasia. Firstly, we measured body weight and prostate weight to assess any differences between *Itln-1*^*−/−*^ mice and WT littermates (Fig. 1B–E). In 5-month-old mice, the prostate tissues of *Itln-1*^*−/−*^ mice had a larger size than those of WT littermates (Fig. 1B). Although there was no significant difference between the body weight (Fig. 1C) and prostate weight (Fig. 1D), a notable difference was observed in the prostate index (prostate weight/body weight, mg/g; Fig. 1E), which considered as a critical indicator of BPH development. Additionally, compared to 10-month-old WT mice, the body weight, prostate weight, and prostate index of 10-month-old *Itln-1*^*−/−*^ mice increased by approximately 14.7% (*P* = 0.0093; Fig. 1C), approximately 29.6% (*P* = 0.0015; Fig. 1D), and approximately 1.18-fold (*P* = 0.0057; Fig. 1E), respectively. This observation strongly suggested that omentin-1 deficiency might play a role in the development of BPH.

BPH is a common prostate pathology that often requires clinical evaluation and follow-up (Alkan et al. 2016). Some BPH patients may exhibit elevated serum levels of prostate-specific acid phosphatase (PAP), which is a glycoprotein secreted by the prostate (Hakalahti et al. 1993; Sarwar et al. 2017; Zou et al. 2017). Compared to WT littermates, 5-month-old *Itln-1*^*−/−*^ mice showed an approximately 23.8% increase in serum PAP concentration (Fig. 1F). Furthermore, histomorphology analysis revealed that the glandular area in *Itln-1*^*−/−*^ mice increased by approximately 1.9-fold (*P* = 0.0003; Fig. 1G, H). As shown in Fig. 1I, our analysis revealed a decreasing trend in the stromal/epithelial ratio in the 10 M *Itln-1*^*−/−*^ group compared to the 10 M WT group, but the difference was not statistically significant. Interestingly, a notable decrease in the stromal/epithelial ratio (about 1.6-fold) was observed in the 10 M *Itln-1*^*−/−*^ group compared with the 5 M *Itln-1*^*−/−*^ group (*P* = 0.0062). Both are characteristic features of BPH, further suggesting the crucial role of omentin-1 in maintaining normal prostate physiology.

### Itln-1 deficiency induces inflammation in the prostate

**Fig. 2:**
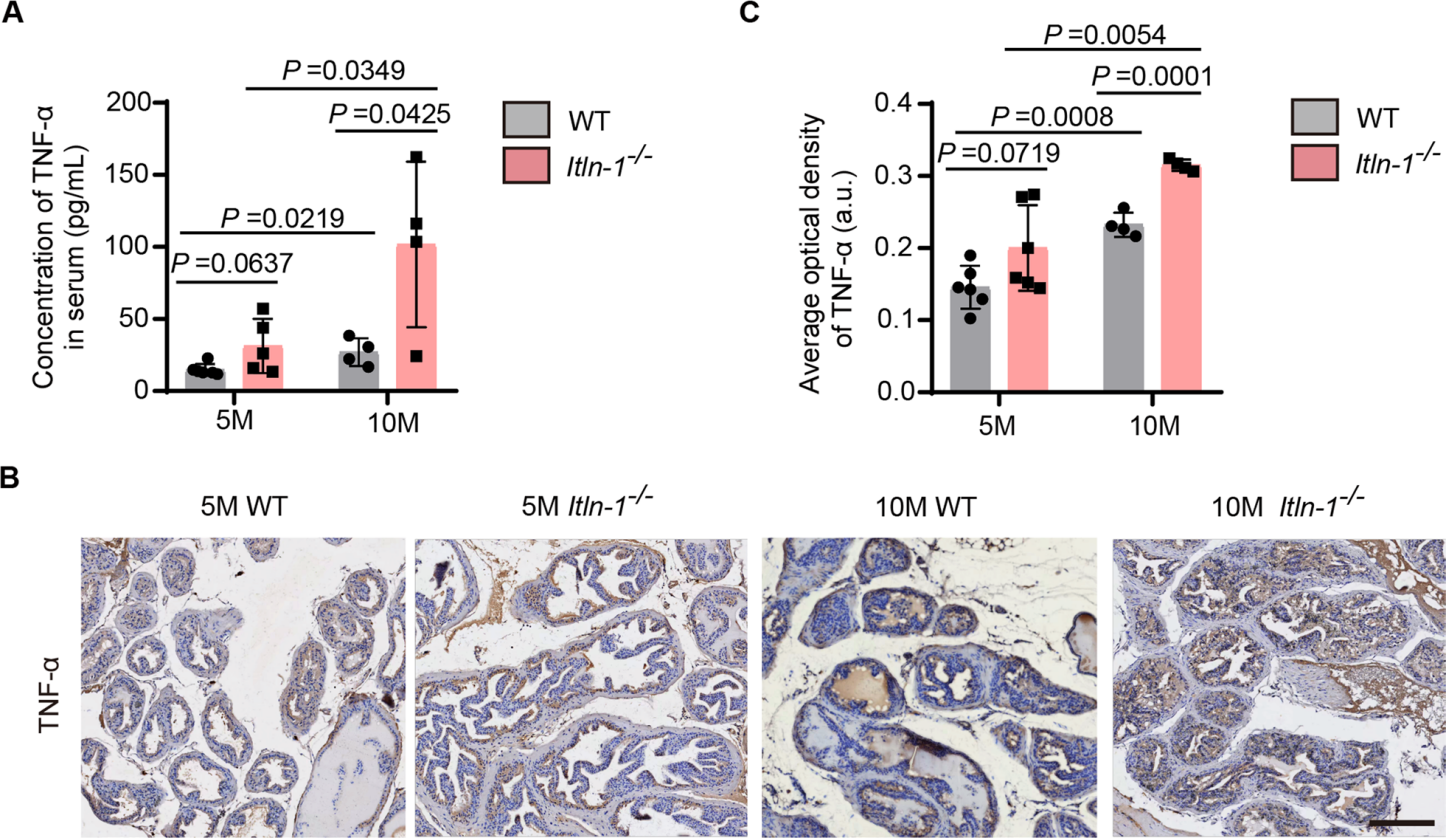
*Itln-1* deficiency induces inflammation in the prostate. **A** Serum levels of TNF-α in WT and *Itln-1*^*−/−*^ mice measured by ELISA. n = 5 for 5-month-old mice, n = 4 for 10-month-old mice, two-way ANOVA. **B**, **C** Representative images (**B**) and average optical density (**C**) of TNF-α staining in the cytoplasm of luminal epithelium in prostate tissues from WT and *Itln-1*^*−/−*^ mice, observed under 200-fold magnification. Scale bar: 200 μm. n = 6 for 5-month-old mice, n = 4 for 10-month-old mice, two-way ANOVA. The values of the individual statistical significances and statistical comparisons were indicated in the figure

Inflammation is a critical factor in the pathogenesis of BPH, contributing to the recruitment of immune cells and the secretion of proinflammatory cytokines, which ultimately lead to prostate gland dysfunction and structural deformities. Thus, our study also revealed that omentin-1 deficiency was associated with an increased inflammation response in the prostate tissue of *Itln-1*^*−/−*^ mice. To further investigate this phenomenon, we measured the serum concentrations of tumor necrosis factor-α (TNF-α), a proinflammatory cytokine that played a crucial role in the regulation of immune responses, using enzyme-linked immunosorbent assay (ELISA). The results clearly showed the serum TNF-α levels in 10-month-old *Itln-1*^*−/−*^ mice increased approximately 3.8-fold compared to 10-month-old WT mice (*P* = 0.0425; Fig. 2A), suggesting a possible link between omentin-1 deficiency and systemic inflammation. Furthermore, we performed immunohistochemical staining on prostate tissue sections (Fig. 2B), which revealed an approximately 40% increase in TNF-α staining in the epithelial cells of *Itln-1*^*−/−*^ mice (*P* = 0.0001; Fig. 2C).

### Omentin-1 suppresses TNF-α-activated macrophage-mediated proliferation of BPH-1 cells

**Fig. 3:**
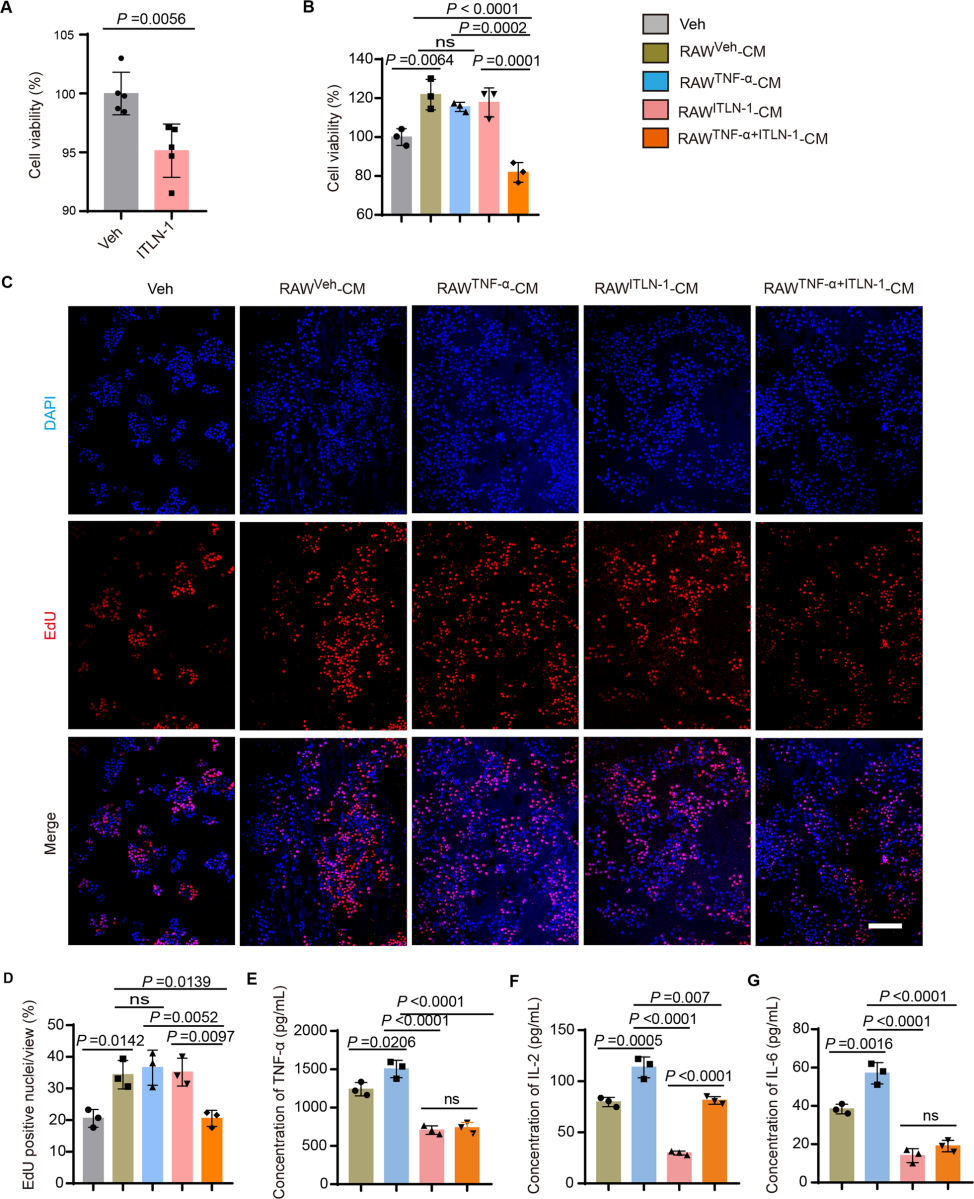
Omentin-1 suppresses TNF-α-activated macrophage-mediated proliferation of BPH-1 cells. **A** The effect of ITLN-1 protein on BPH-1 cell viability, as measured by CCK8 assay. n = 5, t-test. **B** Cell viability of BPH-1 cells after indicated different treatments. n = 3, one-way ANOVA. **C**, **D** Representative images of EdU staining (**C**) and percentage of EdU positive nuclei in per field (**D**) of BPH-1 cells after indicated different treatments, observed under 100-fold magnification. Scale bar: 100 μm. Data represented as mean ± SEM. n = 3, one-way ANOVA. **E**–**G** The concentration of TNF-α (**E**), IL-2 (**F**), and IL-6 (**G**) in the concentrated supernatant of RAW264.7 macrophages stimulated with Vehicle, TNF-α, ITLN-1, or TNF-α + ITLN-1. n = 3, one-way ANOVA. The values of the individual statistical significances and statistical comparisons were indicated in the figure

In addition to studying the effects of omentin-1 deficiency on prostate inflammation, we also investigated the impact of omentin-1 protein on a human benign prostatic hyperplasia cell line BPH-1. BPH-1 cells are a widely used cell line to study BPH pathogenesis. By incubating BPH-1 cells in fresh culture medium supplemented with recombinant ITLN-1 or an equal volume of phosphate buffered saline (PBS) as a vehicle, we were able to assess the potential effects of ITLN-1 on cell proliferation of BPH-1. The results showed that ITLN-1 had an inhibitory effect on BPH-1 cell proliferation (*P* = 0.0056; Fig. 3A). This finding is noteworthy and suggests that omentin-1 may have therapeutic potential in BPH management.

To better understand the potential indirect effects of omentin-1 on BPH proliferation, we conducted further investigations into the role of inflammation in BPH development. We hypothesized that omentin-1 may be able to inhibit the proliferation-stimulating factors secreted from activated macrophages, thereby indirectly regulating BPH-1 cell proliferation. Murine macrophage/monocyte cell line RAW264.7 is commonly used to investigate the role of macrophages in the inflammatory response (Xu et al. 2021; Zou et al. 2017). We established an inflammatory macrophage model by exposing RAW264.7 to recombinant TNF-α for 30 min. The normal and inflammatory RAW264.7 cells were then treated with recombinant ITLN-1, and these cell culture conditioned media (CM) were collected and concentrated for further use.

Then, BPH-1 cells were cultured in RPMI 1640 medium supplemented with the concentrated CM from RAW264.7 macrophages that has been stimulated by vehicle (Veh), TNF-α, ITLN-1, or TNF-α + ITLN-1. Under a light microscope and cell viability assay, we analyzed the effects of these different CM treatments on BPH-1 cell proliferation. Our results revealed that the concentrated CM from Veh- or TNF-α-stimulated macrophages (RAW^Veh^ − CM or RAW^TNF−α^ − CM) increased the viability of BPH-1 cells by approximately 20% (*P* = 0.0064 or 0.0497; Fig. 3B). Interestingly, compared to RAW^TNF−α^ − CM group, the viability of BPH-1 cells decreased by approximately 25% when treated with RAW^TNF−α +ITLN−1^ − CM (*P* = 0.0002; Fig. 3B). This suggested that the effects of omentin-1 on BPH-1 cell proliferation were highly dependent on the presence or absence of inflammatory mediators. To further confirm our findings, we also used 5-ethynyl-2′-deoxyuridine (EdU) fluorescent staining to examine BPH-1 cells proliferation. The results of this analysis were consistent with the previous findings, further validating the potential role of omentin-1 in regulating BPH-1 cell proliferation (Fig. 3C, D). To verify the hypothesis of ITLN-1 inhibition of macrophage inflammatory cytokine release, we further examined the levels of inflammatory factors in the concentrated supernatant of RAW264.7 macrophages stimulated with Veh, TNF-α, ITLN-1, or TNF-α + ITLN-1 using dedicated ELISA kits. As shown in Fig. 3E–G, TNF-α, IL-2, and IL-6 levels in the CM of RAW264.7 cells induced with TNF-α (RAW^TNF−α^ − CM) were elevated by approximately 21% (*P* = 0.0206), 43% (*P* = 0.0005), and 50% (*P* = 0.0016) compared to the control group (RAW^Veh^ − CM). However, treatment with ITLN-1 (RAW^TNF−α +ITLN−1^ − CM) resulted in a notable decrease in these cytokine levels, with reductions of approximately 51% for TNF-α (*P* < 0.0001), 28% for IL-2 (*P* = 0.007), and 66% for IL-6 (*P* < 0.0001).

### Omentin-1 effectively attenuates testosterone propionate-induced BPH

**Fig. 4:**
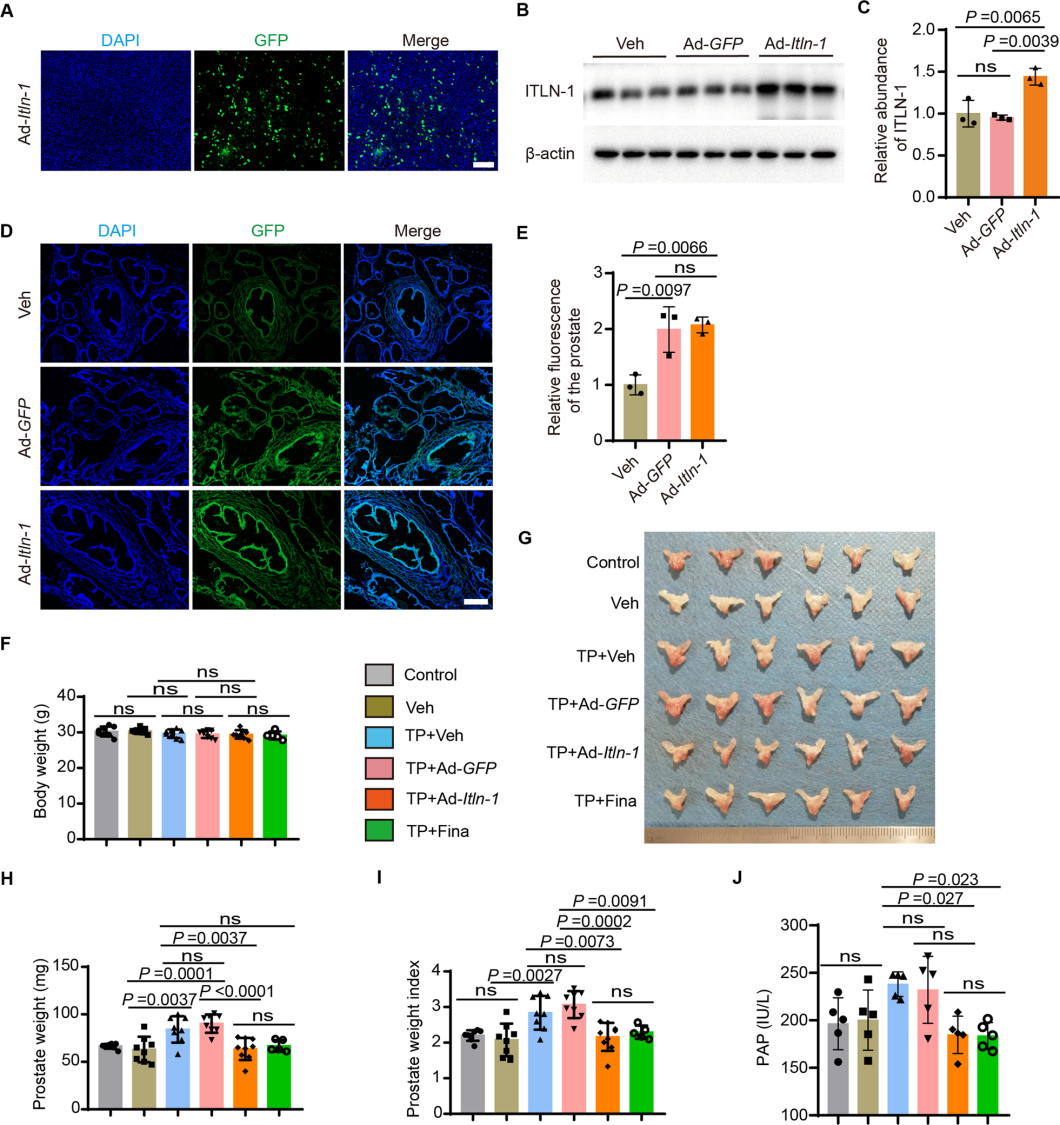
Omentin-1 effectively attenuates testosterone propionate-induced BPH. **A** Representative immunofluorescence images showing the expression of GFP in BPH-1 cells after intervention of Ad-*Itln-1*. Scale bar: 100 μm. **B**, **C** Expression of ITLN-1 protein in prostate samples detected by western blot (**B**) and relative abundance of ITLN-1 in prostate samples (**C**). **D**, **E** Representative fluorescence images and relative fluorescence intensity of prostate tissue after tail vein injection of Ad-*GFP* or Ad-*Itln*-1. Scale bar: 200 μm. n = 3, one-way ANOVA. **F**–**I** body weight at end point (**F**), gross anatomy of prostate tissues (**G**), prostate weight (**H**), and prostate index (**I**) of the mice after indicated different treatments. n = 8, one-way ANOVA. **J** Serum levels of PAP of the mice after indicated different treatments. n = 6, one-way ANOVA. The values of the individual statistical significances and statistical comparisons were indicated in the figure

BPH could be modelled by administering supraphysiological doses of exogenous hormones, such as testosterone propionate (TP), to increase the size of the prostate gland in male mice (Gong et al. 2023). Therefore, we administered daily subcutaneous injections of TP or corn oil to 2-month-old male mice for 28 days. Then, we explored the potential therapeutic effects of omentin-1 in treating TP induced BPH in mice. To confirm the overexpression efficiency of Ad-*Itln-1*, this omentin-1 overexpressing adenovirus was transfected into BPH-1 cells and assessed the GFP intensity by fluorescence staining (Fig. 4A). ITLN-1 overexpression was also verified in the target organs of our animal model. Specifically, we administered adenovirus overexpressing ITLN-1 (Ad-*Itln-1*) or a negative control adenovirus (Ad-*GFP*) via the tail vein into mice at a dose of 1 × 10^8^ PFU per mouse. Tissue samples from the prostate and small intestine were collected 14 days post-injection for analysis. Western blot analysis revealed a significant increase in ITLN-1 expression in the prostate and intestine tissues, with approximately 1.5-fold (*P* = 0.0039; Fig. 4B, C) and 1.7-fold (*P* = 0.0017; Additional file 1: Figure S1A, B) higher levels than the control virus, respectively. Additionally, fluorescence microscopy confirmed the enhanced transfection efficiency in these tissues, with approximately twofold (*P* = 0.0066; Fig. 4D, E) and 2.2-fold (*P* = 0.0075; Additional file 1: Figure S2A, B) increases in the prostate and intestine, respectively.

The mice were given intravenous administration of vehicle (PBS), adenovirus encoding omentin-1 (Ad-*Itln-1*), control adenovirus encoding GFP (Ad-*GFP*), or intraperitoneal injection of the clinical first-line drug finasteride (Fina) as a positive control, respectively. During the 28 days administration period, there was no significant change in the body weight of the mice under different treatments, except for the Fina treated group, which showed a decreasing trend (Fig. 4F). We then proceeded to evaluate the potential therapeutic effects of omentin-1 by analyzing the prostate weight and prostate index in each treatment group. After 28 days of administration, compared to the vehicle (corn oil-treated) group, the prostate weight and prostate index of the TP-treated group increased by approximately 35% (*P* = 0.0037) and 38% (*P* = 0.0027), respectively (Fig. 4G–I), indicating that the BPH model was successfully established. However, after TP + Ad-*Itln-1* treatment, the prostate weight and prostate index decreased by approximately 23.8% (*P* = 0.0037) and 23.6% (*P* = 0.0073) compared to the TP + Veh group, respectively (Fig. 4G–I), while treatment with TP + Ad-*GFP* or TP + Veh had no significant effect (Fig. 4G–I). In addition, there was no significant difference between the TP + Ad-*Itln-1* and TP + Fina groups (Fig4G–I). These results suggested that omentin-1 might prevent the development of BPH by inhibiting TP-induced prostatic hypertrophy. Moreover, we also investigated the levels of PAP in the serum of the different treatment groups. Compared with the control group, the serum levels of PAP in mice in the TP group showed an elevated trend (Fig. 4J). However, when mice were treated with Ad-*Itln-1*, which led to overexpression of omentin-1, the upregulation of PAP levels was reduced, and the effect of Fina was comparable to that of *Itln-1*. (Fig. 4J). This finding suggested that omentin-1 overexpression might have a protective effect against the development and progression of BPH.

### Omentin-1 tones down histomorphological features of BPH

**Fig. 5:**
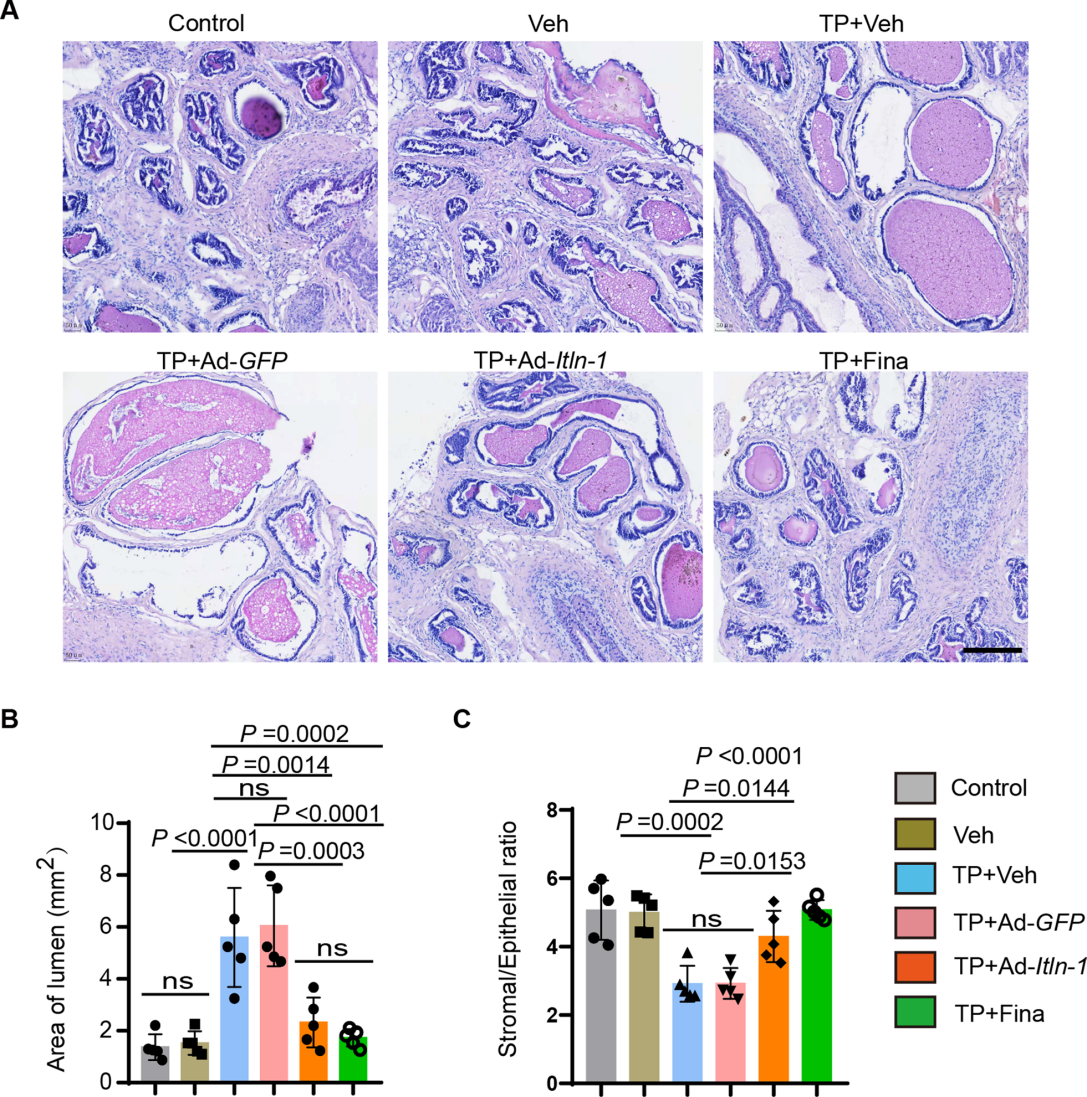
Omentin-1 alleviates histomorphological features of BPH. **A** Representative images of H&E staining of prostate tissues from different treated mice, observed under 200-fold magnification. Scale bar: 200 μm. **B**, **C** Area of lumens (**B**) and stromal/epithelial ratio (**C**) of different treated mice, respectively. n = 5, one-way ANOVA. The values of the individual statistical significances and statistical comparisons were indicated in the figure

Histomorphology is a valuable tool for assessing the efficacy of BPH treatments and enables the identification of structural changes in the prostate tissue. Thus, we also examined the histomorphology of the prostate samples from the different treatment groups to further confirm the effects of omentin-1 treatment. Compared to the control group, the prostate samples from the TP + Ad-*GFP* treated group exhibited an approximately 4.9-fold enlargement in glandular area (*P* < 0.0001; Fig. 5A, B), and a decrease of approximately 58% in stromal/epithelial ratio (*P* = 0.0002; Fig. 5C), both characteristic of BPH. Compared to the TP + Ad-*GFP* treated group, the samples from the TP + Ad-*Itln-1* treated group demonstrated significant improvement in prostate tissue morphology, including a reduction in glandular area by approximately 38% (*P* = 0.0003; Fig. 5A, B), and an increase in stroma/epithelium ratio by approximately 47% (*P* = 0.0153; Fig. 5C).

### Omentin-1 prevents inflammatory prostatic hyperplasia induced by testosterone propionate

**Fig. 6:**
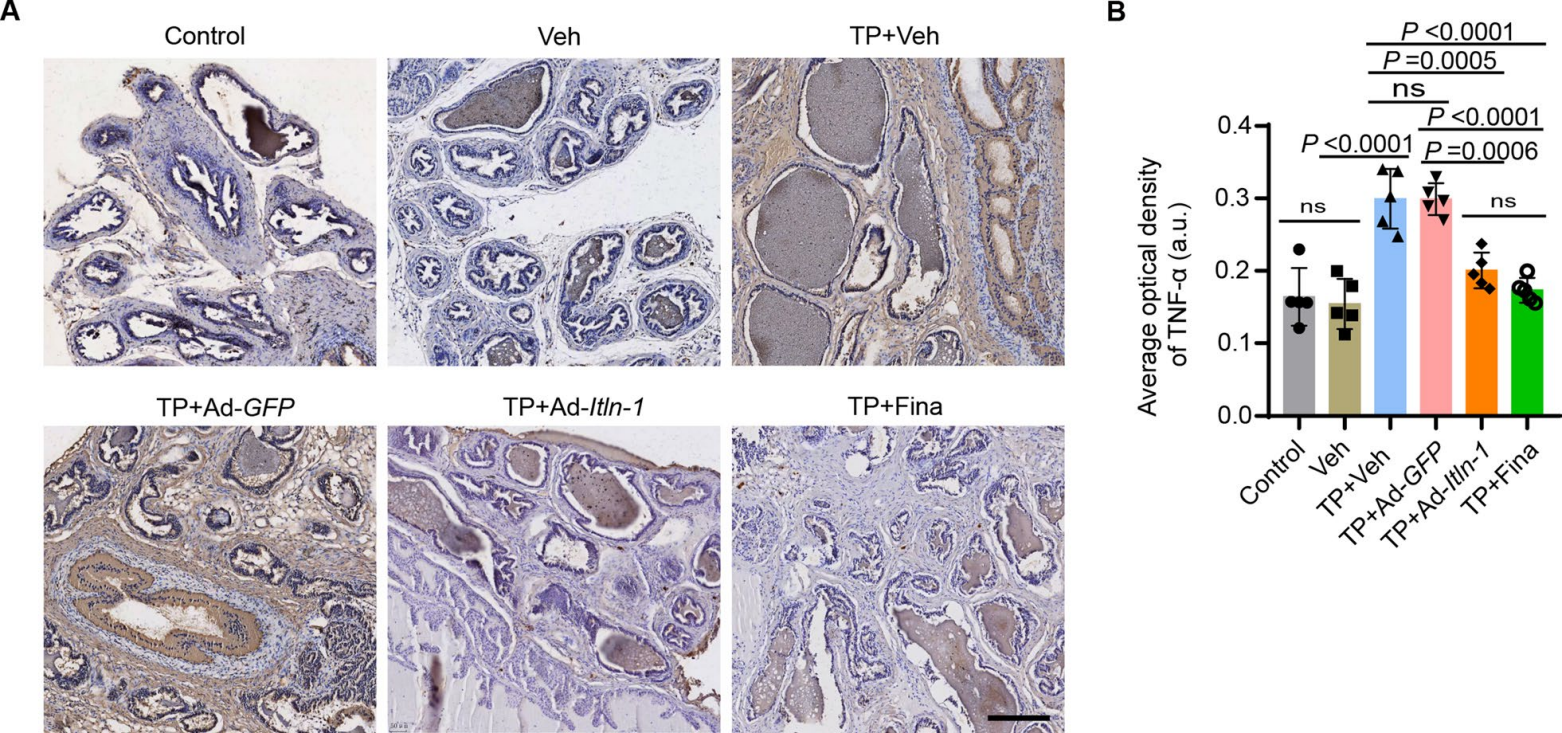
Omentin-1 prevents inflammatory prostatic hyperplasia induced by testosterone propionate. **A**, **B** Representative images (**A**) and average optical density (**B**) of TNF-α staining in the cytoplasm of luminal epithelium in prostate tissues from different treated mice, observed under 200-fold magnification. Scale bar: 200 μm. n = 5, one-way ANOVA. The values of the individual statistical significances and statistical comparisons were indicated in the figure

Furthermore, immunohistochemistry staining was performed to evaluate the expression levels of inflammatory mediators in the prostate tissues of different treated mice. The results showed that, compared to the control group, the expression of TNF-α in the prostate tissue of TP-treated mice increased by approximately 1.94-fold (*P* < 0.0001; Fig. 6A, B). This result is consistent with previous studies that have demonstrated the role of inflammation in the pathogenesis of BPH (Wang et al. 2021). In contrast, the level of TNF-α in the prostate tissue of mice treated with TP + Ad-*Itln-1* decreased by approximately 31% (*P* = 0.0006) compared to TP + Ad-*GFP*, with no significant difference between the TP + Ad-*Itln-1* group and the TP + Fina group (Fig. 6A, B).

## Discussion

Our study provided significant insights into the potential therapeutic applications of omentin-1 for BPH. In our investigation, prostatic hyperplasia, higher prostate weight, and prostate inflammation levels were observed to be more severe in omentin-1 knockout mice compared to WT mice, suggesting a critical role for omentin-1 deficiency in the pathogenesis of BPH. The increased PAP levels and enlarged prostate glandular area in omentin-1 knockout mice provided in vivo evidence of a potential connection between omentin-1 and the development of BPH. In contrast, in a TP-induced BPH mouse model, adenovirus-mediated overexpression of ITLN-1 resulted in reduced prostate weight and local inflammation. These findings provide the first in vivo evidence supporting the potential role of ITLN-1 in the physiology and pathogenesis of BPH. Significantly, our results suggest that ITLN-1 deficiency may contribute to the development of BPH, potentially through the upregulation of proinflammatory factors and subsequent prostate inflammation.

Further, our study indicates that ITLN-1 might directly inhibit prostate epithelial cell proliferation and indirectly regulate cell proliferation through suppressing macrophage-mediated inflammatory responses. This dual mechanism of action underlines the therapeutic potential of ITLN-1 in managing BPH. Our findings also highlight the protective effect of ITLN-1 against TP-induced prostate hypertrophy, as evidenced by the reduction in prostate weight and PAP levels.

Additionally, histomorphological examination of prostate samples from the study indicates that ITLN-1 treatment could effectively mitigate BPH-associated histomorphological changes. This supports the potential of ITLN-1 as a valuable therapeutic agent for addressing BPH. These findings suggest that omentin-1 hold potential as a therapeutic target for BPH, especially for patients who may not tolerate or respond well to currently available medications.

Adipokines regulate various biochemical processes, including inflammatory response, blood pressure, fibrinolysis, and immunity. Omentin-1, which is mainly produced by adipose tissue, plays a critical role in regulating a variety of physiological processes (Watanabe et al. 2017). Although adipokines such as adiponectin and leptin are known to have positive and negative effects on BPH, respectively, the significance of omentin-1, a novel adipokine, in BPH is still underexplored. Omentin-1 is considered a beneficial adipokine, similar to adiponectin, that is involved in various physiological processes, such as energy expenditure, carbohydrate and lipid metabolism, bone metabolism, inflammatory response, and immunity (Rao et al. 2018) (Qi et al. 2016). Our study contributes to the increasing evidence that suggests adipokines, particularly omentin-1, may have a beneficial role in BPH management.

Metabolic syndrome is a group of metabolic disorders that includes central obesity, dyslipidemia, hypertension, insulin resistance with compensatory hyperinsulinemia, and glucose intolerance. Studies have shown that metabolic syndrome and its related comorbidities, such as sex steroid alterations and low-grade inflammation, have been related to BPH development and progression (Vignozzi et al. 2013; Wang et al. 2012). Lifestyle modifications and exercise, which are known to ameliorate metabolic syndrome and reduce the risk of developing BPH, are also associated with increased omentin-1 expression (Moyad and Lowe 2008). This correlation suggests that omentin-1 may play a role in mediating the beneficial effects of these interventions on BPH.

Chronic low-grade inflammation is a central pathogenic factor in the development of metabolic syndrome components (Nickel et al. 2016). Furthermore, recent clinical and preclinical evidence has confirmed that chronic inflammation also contributes to the development of BPH (Luo et al. 2020; Park et al. 2021). Clinical studies suggest that omentin-1, with its anti-inflammatory properties, may serve as a biomarker for inflammatory diseases and metabolic syndrome (Tan et al. 2010). Conversely, restoring omentin-1 levels through therapeutic strategies, such as targeted omentin-1 administration, may provide a viable approach for preventing or treating BPH in obese individuals or those with low levels of this protein. The therapeutic potential of omentin-1 in BPH is closely linked to its anti-inflammatory properties, which are similar to those of adiponectin.

In this study, we measured PAP, which is produced by mature prostate epithelial cells, as an indicator of prostate hypertrophy. The elevated serum levels of PAP can indicate several diseases, including prostate cancer (Sarwar et al. 2017). Notably, studies have demonstrated that the PAP content and PAP-encoding mRNA levels are significantly higher in BPH tissue than in malignant prostatic hyperplasia tissue (Hakalahti et al. 1993). Furthermore, our findings are supported by the elevated PAP levels observed in our TP-induced BPH mouse model (Zou et al. 2017).

However, our study has several limitations. Further studies are necessary to fully elucidate the mechanisms by which omentin-1 inhibits BPH and to explore its potential clinical applications. Future investigations should examine the effects of omentin-1 on human BPH tissues both in vitro and in vivo. Additionally, clinical trials are needed to evaluate the safety and efficacy of omentin-1 as a treatment option for BPH patients. Additionally, exploring the interplay between lifestyle interventions, omentin-1 expression, and BPH progression could offer valuable insights into non-pharmacological BPH management strategies. Ultimately, understanding the molecular mechanisms of omentin-1's influence on prostate health is crucial for developing targeted therapies, paving the way for innovative and personalized treatments for BPH and associated conditions.

## Conclusion

In conclusion, our results indicate that targeting ITLN-1 could be an effective treatment strategy for BPH, as ITLN-1 plays a crucial role in suppressing local inflammation in the prostate gland. This research opens up new avenues for the development of anti-inflammatory strategies in the treatment of BPH.

## Materials and methods

### Cell culture

Mouse leukemic macrophage cell line RAW264.7 (Cat. No. CL-0190) was purchased from Procell Life Science & Technology. It was cultured in high-glucose Dulbecco’s Modified Eagle’s Medium (DMEM; Cat. No. 11965092, Gibco) supplemented with 10% heat-inactivated fetal bovine serum (FBS; Cat. No. SA301.02, CellMax) and 1% Penicillin–Streptomycin (P/S; Cat. No. P1400, Solarbio). Human prostate hyperplasia cell line BPH-1, kindly provided by Prof. Long Wang from The Third Xiangya Hospital of Central South University in Changsha, China, was cultured in RPMI 1640 medium (Cat. No. C3010-0500, VivaCell) supplemented with 10% FBS and 1% PS. All cells were maintained at 37 °C in a fully humidified atmosphere of 95% air and 5% CO_2_.

### Preparation of conditioned culture medium from inflammatory macrophages

We induced inflammation in RAW264.7 macrophages by using 10 ng mL^−1^ of pro-inflammatory cytokine TNF-α (Cat. No. 300-01A, PeproTech), as our previously reported (Deng et al. 2022). Subsequently, the residual culture medium was replaced and the cells were incubated in fresh CM supplemented with either 300 ng mL^−1^ of recombinant ITLN-1 protein (Cat. No. abx067296, Abbexa) or an equal volume of PBS for 24 h. After incubation, the conditional medium (CM) from inactivated (treated with Veh; RAW^Veh^ − CM), TNF-α-activated (RAW^TNF−α^ − CM), ITLN-1-treated (RAW^ITLN−1^ − CM), or TNF-α + ITLN-1-treated (RAW^TNF−α +ITLN−1^ − CM) RAW264.7 macrophages were collected and subjected to centrifugation at 2,000 × g for 10 min to remove dead cells and cellular debris. The supernatant was then collected and stored at − 80 °C or used for downstream experiments.

### Cell counting kit-8 assay

Cell proliferation in BPH-1 cells was assessed by the Cell Counting Kit-8 (CCK-8; Cat. No. C0005, TargetMol) following to the manufacturer’s protocol. To evaluate the effect of omentin-1 on BPH-1 cells, the cells were firstly seeded in a 96-well plate at a density of 2 × 10^3^ cells per well, and cultured for an additional 48 h. The cells were then subjected to PBS or ITLN-1 protein (300 ng mL^−1^) treatment. To assess the effect of the conditional medium (CM) of RAW264.7 cells intervened by ITLN-1 on BPH-1 cells, we treated BPH-1 cells with the following medium: Veh, RAW^Veh^ − CM, RAW^TNF−α^ − CM, RAW^ITLN−1^ − CM, or RAW^TNF−α +ITLN−1^ − CM, respectively. After incubation at 37 °C for 48 h, the medium in each well was replaced with 90 µL fresh medium and 10 µL CCK-8 reagent, followed by incubation at 37 °C for 2 h. Finally, the absorbance was measured using a microplate reader (Varioskan LUX, Thermo Scientific) with a wavelength of 450 nm. The obtained OD value was used to calculate the final results.

### EdU cell imaging assay

The cell proliferation in BPH-1 cells was also assessed by Yefluor 594 Click-iT EdU Imaging Kits (Cat. No. 40276ES60, Yeasen). For EdU imaging assay, BPH-1 cells were seeded into a 24-well plate slide and treated them with different conditions, including: Veh, RAW^Veh^ − CM, RAW^TNF−α^ − CM, RAW^ITLN−1^ − CM, or RAW^TNF−α +ITLN−1^ − CM, respectively. Subsequently, the cells were incubated with the EdU reagent according to the manufacturer's instructions. The EdU reagent is a thymidine analogue that incorporates into the DNA of proliferating cells and can be detected by fluorescence microscopy. After a 2-h incubation, the cells were fixed in 4% paraformaldehyde and stained with 4′,6-diamidino-2-phenylindole (DAPI; Cat. No. H-1200, Vectorlabs) to visualize nuclei. Finally, the fluorescent images were acquired using a Zeiss ApoTome fluorescence microscope. The percentage of EdU positive cells was quantified for different groups.

### Animals and treatments

To ensure the welfare of the animals, they were housed in a controlled environment with appropriate temperature (22 ± 3 °C), humidity (50 ± 20%), and lighting (12/12 h light-darkness cycle) conditions. Additionally, all animals had access to sufficient food and water sources throughout the experiment.

To evaluate the prostate states under omentin-1 deficiency, 5-month-old and 10-month-old C57BL/6 male *Itln-1*^*−/−*^ mice and their WT littermates were used for further analyses. The *Itln-1*^*−/−*^ mice were constructed by Cyagen Biosciences. The depletion of *Itln-1* was verified by RT-PCR on collected small intestines from both *Itln-1*^*−/−*^ mice and their WT littermates.

To investigate the therapeutic effect of omentin-1 on BPH, the 2-month-old male C57BL/6 WT mice were used to induce experimental BPH models by continuous intraperitoneal injection of testosterone propionate (TP, dissolved in 100 μL of corn oil; Cat. No. C805618, Macklin) at the dosage of 5 mg per Kg body weight each day, or equal volume of corn oil as a control. Mice were randomly divided into the following 6 groups, including control, corn oil, TP, TP + Ad-*Itln-1*, TP + Ad-*GFP*, TP + Fina groups (n = 8 per group). Ad-*Itln-1* or Ad-*GFP* at the dose of 1 × 10^8^ plaque-forming units (PFU) were intravenously injected into the tail vein of mice for twice a week. Body weight of mice was recorded weekly to adjust the TP dosage. After four weeks treatment, all mice were sacrificed to collect blood, prostate, and small intestine samples immediately after euthanasia. The serum samples were obtained by centrifugation at 1500 rpm for 15 min and stored at − 80 °C for further analyses. The prostate index was calculated by dividing the prostate wet weight (mg) by the body weight (g) of the mice. Histological analysis of the prostate tissues was performed after immersing them in 4% PFA or freezing them at − 80 °C.

### Histomorphometry and immunohistochemical analyses

Prostate tissues were embedded in paraffin after dehydration in an increased gradient ethanol solution. We then processed 5 μm-thick sections of the prostate and performed histomorphometric and immunohistochemical analysis to gain the information of BPH pathogenesis. For histomorphological analysis, hematoxylin–eosin (H&E; Cat. No. G1005, Servicebio) staining was conducted to examine the samples. For immunohistochemical analysis, the sections were incubated with primary antibody against TNF-α (1:200 dilution; Cat. No. 17590, Proteintech) overnight at 4 °C. Subsequently, the slices were incubated with HRP-conjugated secondary antibody (Cat. No. 511203, ZenBio) for 1 h at room temperature. Histomorphometric and immunohistochemical analysis were observed under an optical microscope (Olympus CX31). Positive staining cells or relative staining area were measured in three random visual fields per section using Image-Pro Plus 6.0 software.

### ELISA analysis

The levels of prostatic acid phosphatase (PAP), TNF-α, IL-2, and IL-6were evaluated using a mouse PAP ELISA kit (Cat. No. JM-02750M2, JingMei Biotechnology),TNF-α ELISA kit (Cat. No. 70-EK282/4-96, Elabscience), IL-2 ELISA kit (Cat. No. 70-EK202/2-96, Elabscience) and IL-6 ELISA kit (Cat. No. 70-EK206/3-96, Elabscience). All ELISA assays were performed according to the manufacturers’ instructions with strict quality control measures in place.

### Western blot

Tissue grinding is performed in RIPA buffer containing 1% protease inhibitor. After a short sonication, the tissue homogenate is centrifuged at 3000×*g* for 20 min (4 °C). Tissue extracts were obtained by collecting the supernatant. Protein concentration was determined using BCA Protein Quantification Kit (Cat. No. E-BC-K318-M, Elabscience). The target proteins were detected by immunoblotting using ITLN-1 antibody (Cat. No. 11770-1-A, Proteintech). The intensity of the bands was quantified using imageJ software.

### qRT-PCR

Total RNAs were isolated from tissues using TRIzol reagent (Cat. No. AG21102, AG), and the concentrations of the RNAs were measured using Varioskan LUX (Thermo Scientific). The All-in-One cDNA Synthesis SuperMix (Cat. No. E0 47, Novoprotein) was used to synthesize cDNA following the manufacture’s protocol. The qRT-PCR analysis was performed on the FTC-3000 real-time PCR system (Funglyn Biotech) using 2 × SYBR Green qRT-PCR Master Mix (Cat. No. 21202, Bimake). Relative gene expression was calculated by the 2^*–△△CT*^ method, with *Gapdh* serving as the reference housekeeping gene.

The primers used for qRT-PCR were designed based on the gene sequences and were as follows: *mus musculus Gapdh*, forward, 5ʹ-CACCATGGAGAAGGCCGGGG-3′, reverse, 5′-GACGGACACATTGGGGGTAG-3′; *Mus musculus Itln-1*, forward, 5′-TTTCCTGCGCACGAAGAA-3′, reverse, 5′-TCATGTCACAGAAGGTCT-3′.

### Statistical analysis

Data were presented as mean ± standard deviation (SD) and statistically analyzed using GraphPad Prism 7.0 software. The sample size (n) for each statistical analysis is specified in the figure legends. Unpaired two-tailed Student’s t-test was used to compare two groups, and one-way analysis of variance (ANOVA) was applied to compare multiple groups, and two-way ANOVA was used when two independent variables, in combination, affect a dependent variable. The difference was considered statistically significant at *P* < 0.05, *P* < 0.01, and *P* < 0.001. NS, not significant.

### Supplementary Information

**Additional file 1: Figure S1.** Efficiency of Ad-*Itln-1* expression in the small intestine detected by western blot. **Figure S2.** Fluorescence assessment of Ad-*Itln-1* transfection efficiency in the small intestine.

## Data Availability

The data that support the findings of this study are available from the corresponding author upon reasonable request.

## Abbreviations

BPH: Benign prostatic hyperplasia
ITLN-1: Intelectin-1
TP: Testosterone propionate
H & E: Hematoxylin and Eosin
TNF-α: Tumor necrosis factor-alpha
IL-2: Interleukin-2
IL-6: Interleukin-6
*Itln-1*^*−/−*^: ITLN-1 knockout
WT: Wild type
RT-qPCR: Reverse transcription quantitative PCR
PAP: Prostate-specific acid phosphatase
ELISA: Enzyme-linked immunosorbent assay
PBS: Phosphate buffered saline
Veh: Vehicle
CM: Cell culture conditioned media
EdU: 5-Ethynyl-2′-deoxyuridine
Ad-*Itln-1*: Adenovirus encoding intelectin-1
Ad-*GFP*: Adenovirus encoding GFP
Fina: Finasteride
PFU: Plaque-forming units
PFA: Polyformaldehyde
SD: Standard deviation
ANOVA: Analysis of variance
