# Supplementary material for: Omentin-1 inhibits the development of benign prostatic hyperplasia by attenuating local inflammation
Source: Mol Med. 2024 Mar 22;30:41. doi: 10.1186/s10020-024-00805-y (PMC10960431; doi:10.1186/s10020-024-00805-y)
Supplement: Supplementary file 1 — Additional file 1: Figure S1. Efficiency of Ad-Itln-1 expression in the small intestine detected by western blot. Figure S2. Fluorescence assessment of Ad-Itln-1 transfection efficiency in the small intestine. [file 10020_2024_805_MOESM1_ESM.docx]

**Additional Information**

**Omentin-1 inhibits the development of benign prostatic hyperplasia by attenuating local inflammation**

Yi-Yi Wang^1,3,4^, Guo-Qiang Zhu^1,3,4^, Kun Xia^5^, Hong-Bo Zeng^2^, Yun-Hui He^2^, Hui Xie^1,3,4^, Zhen-Xing Wang^1,3,4*^, Ran Xu^2*^

**Affiliations**

^1^Department of Orthopedics, Movement System Injury and Repair Research Center, Xiangya Hospital, Central South University, Changsha, Hunan 410008, China.

^2^Department of Urology, The Second Xiangya Hospital, Central South University, Changsha, Hunan 410011, China.

^3^Hunan Key Laboratory of Angmedicine, Changsha, Hunan 410008, China.

^4^National Clinical Research Center for Geriatric Disorders (Xiangya Hospital), Changsha, Hunan 410008, China.

^5^Department of Orthopedics, People's Hospital of Ningxia Hui Autonomous Region, Yinchuan, Ningxia 750000, China.

**^*^**Corresponding authors: wangzx@csu.edu.cn; xuran@csu.edu.cn.


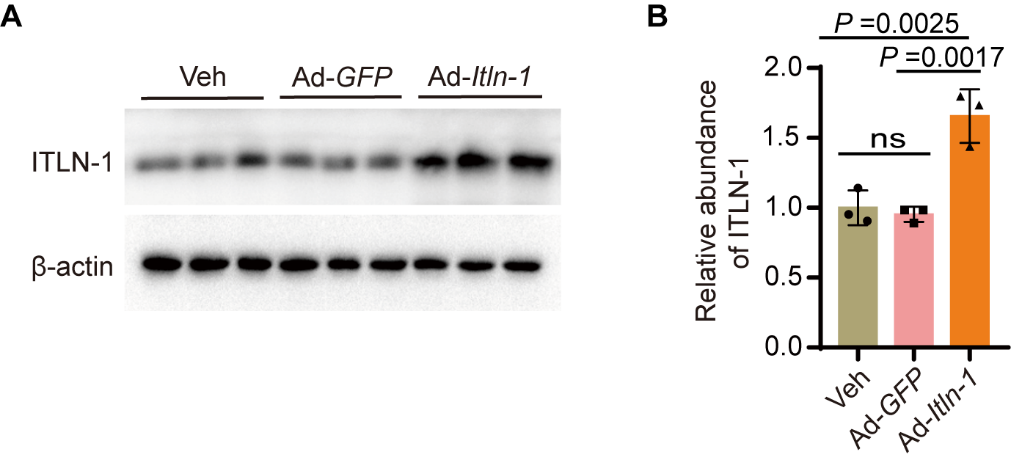


**Figure S1. Efficiency of Ad-*Itln-1* expression in the small intestine detected by western blot.** Expression of ITLN-1 protein in small intestinal samples detected by western blot (A) and relative abundance of ITLN-1 in small intestinal samples (B). n =3, one-way ANOVA. The values of the individual statistical significances and statistical comparisons were indicated in the figure.


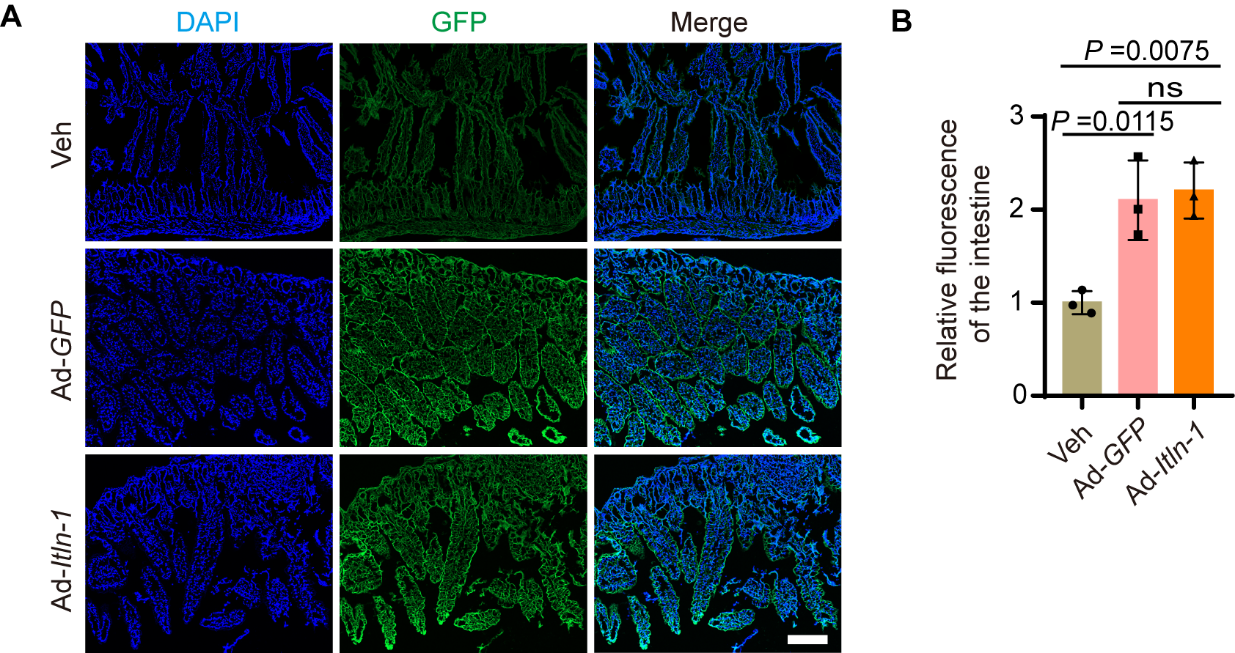


**Figure S2.** **Fluorescence assessment of Ad-*Itln-1* transfection efficiency in the small intestine.** Representative fluorescence images (A) and relative fluorescence intensity of small intestinal samples (B) after tail vein injection of Ad-*GFP* or Ad-*Itln*-1. Scale bar: 200 μm. n =3, one-way ANOVA. The values of the individual statistical significances and statistical comparisons were indicated in the figure.
